# Supplementary material for: Mutant p53s generate pro-invasive niches by influencing exosome podocalyxin levels
Source: Nat Commun. 2018 Nov 29;9:5069. doi: 10.1038/s41467-018-07339-y (PMC6265295; doi:10.1038/s41467-018-07339-y)
Supplement: Supplementary file 1 — Supplementary Information [file 41467_2018_7339_MOESM1_ESM.pdf]

Supplementary Information Novo et al.

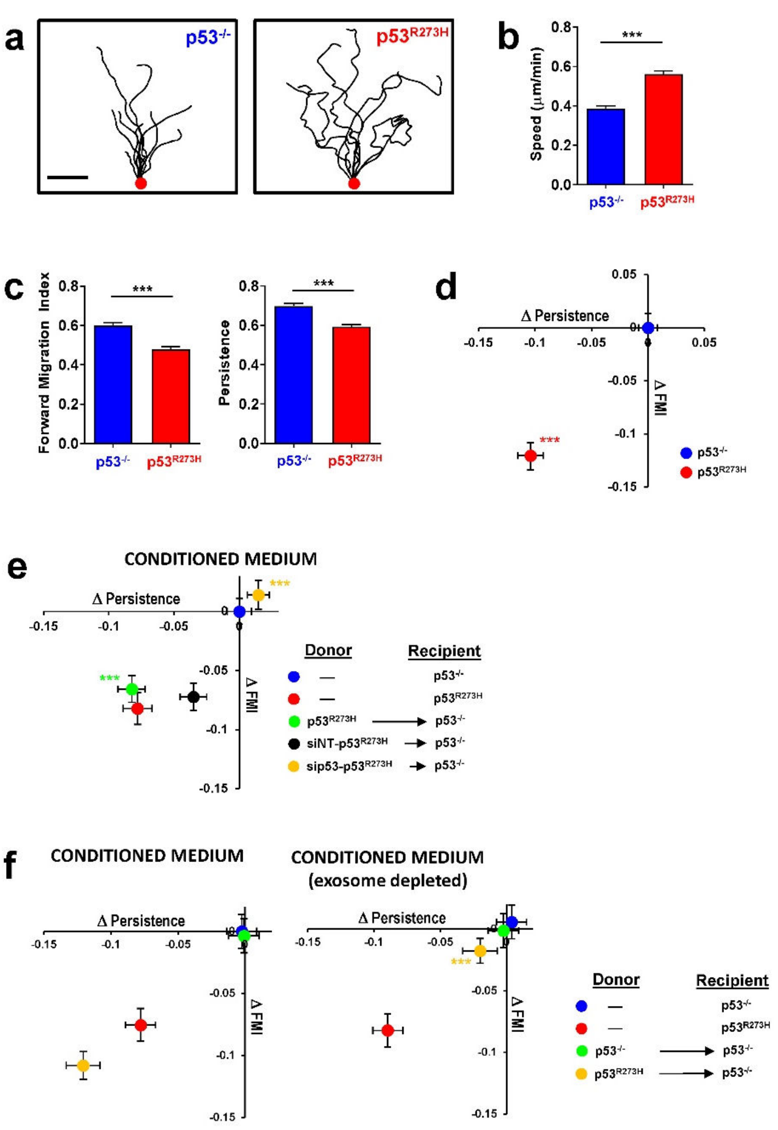

Supplementary Figure 1 Exosomes mediate intercellular transfer of mutant p53's migratory phenotype

(a-d) Confluent monolayers of H1299-p53$^{-/-}$ or H1299-p53^{R273H} cells were wounded with a pipette tip and their migration into these scratch-wounds was monitored by time-lapse microscopy followed by cell tracking. Representative tracks are displayed in (a). Bar, 100 μm. The speed (b), persistence and forward migration index (FMI) (c) of migration into scratch-wounds was determined using cell tracking software. The differences between the migratory persistence and FMI (the ΔPersistence & ΔFMI respectively) of H1299-p53^{R273H} and H1299-p53$^{-/-}$ cells were calculated and are represented graphically in (d). Values are mean ± SEM; *** p<0.001 Mann-Whitney test.

(e, f) Conditioned medium was collected from H1299-p53$^{-/-}$ and H1299-p53^{R273H} 'donor' cells, which were either untransfected or transfected with siRNAs targeting p53 (sip53-p53^{R273H}) or a non-targeting control (siNT-p53^{R273H}). Conditioned medium was then placed onto H1299-p53$^{-/-}$ 'recipient' cells for 72 hr. In the right-hand panel of (f) the conditioned medium was depleted of exosomes by centrifugation at 100,000 g for 70 min prior to being added to recipient cells. Recipient cells were then replated, grown to confluence and wounded, and the ΔPersistence and ΔFMI of migration into scratch-wounds determined as for (d). Values are mean ± SEM; ***yellow versus black and ***green versus blue in (e) is p<0.001; ***yellow versus red in (f) is p<0.001, Mann-Whitney test.

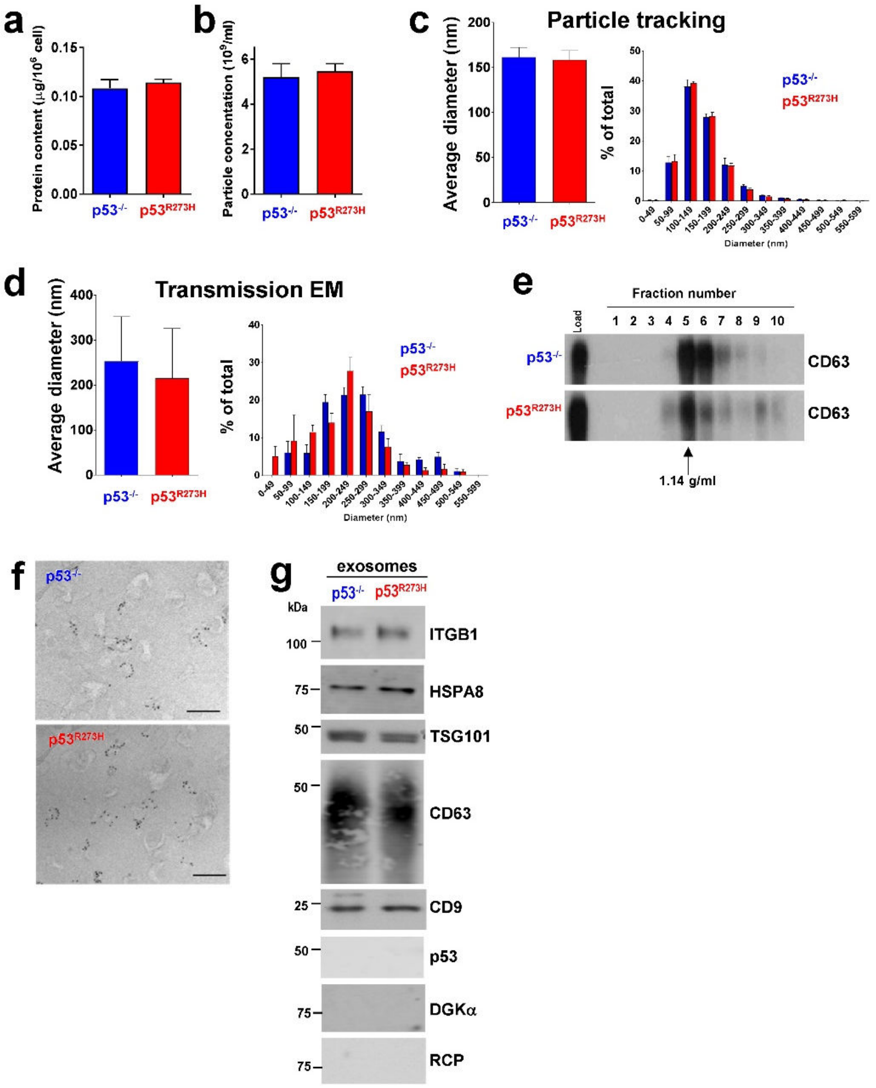

Supplementary Figure 2 Characterisation of exosomes from H1299 cells

Exosomes were purified from H1299-p53$^{-/-}$ and H1299-p53^{R273H} cell conditioned medium using differential centrifugation and the protein (a) and particle (b) concentration, average particle diameter and size distribution (c) of this material were determined using Qubit protein assay and Nanosight particle tracking respectively. The average diameter and size distribution of exosomes from H1299-p53$^{-/-}$ and H1299-p53^{R273H} were also determined using transmission electron microscopy followed by analysis using ImageJ (d). Values are mean ± SEM. Exosome pellets from H1299-p53$^{-/-}$ and H1299-p53^{R273H} cells were characterised using sucrose density gradient centrifugation followed by Western blotting for the exosome marker, CD63 (e). In (f), exosomes were fixed, adsorbed onto nitrocellulose-coated Formvar grids, negatively stained and labelled with anti-CD63 conjugated to 10 nm gold particles. Bar 200 nm. In (g) exosomes from H1299-p53$^{-/-}$ and H1299-p53^{R273H} were purified by differential centrifugation and analysed by western blotting for the presence of established exosome markers and for the presence of p53.

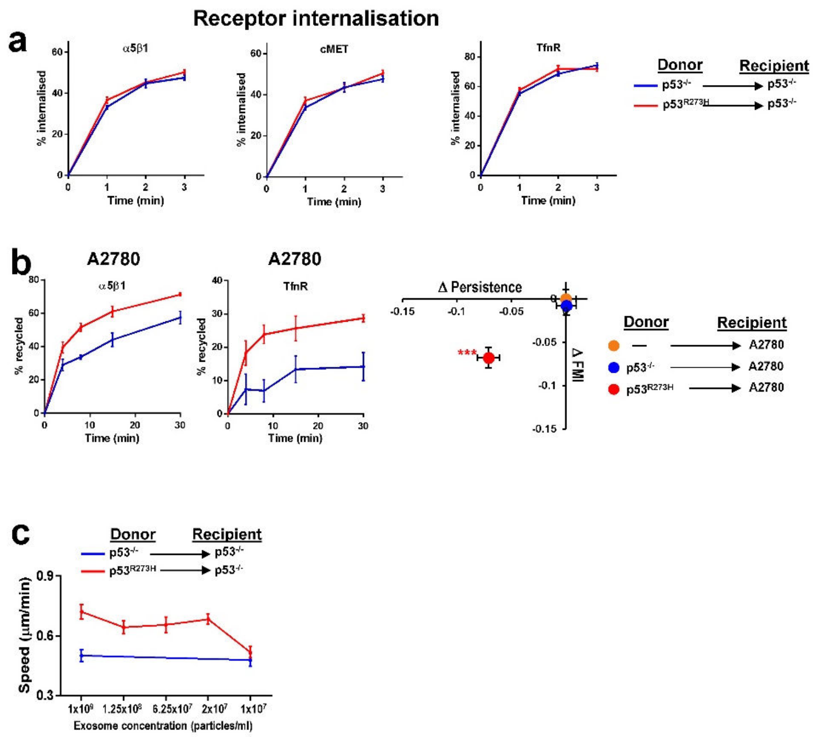

Supplementary Figure 3 Influence of exosomes on internalisation, recycling and cell migration.

(a) H1299-p53$^{-/-}$ ‘recipient’ cells were pre-treated for 72 hr with exosomes collected from H1299-p53$^{-/-}$, or H1299-p53^{R273H} ‘donor’ cells. Recipient cells were then trypsinised and re-plated. 72 hr following re-plating, internalisation of integrin α5β1, cMET and TfnR was determined in the presence of 0.6mM primaquine to inhibit recycling. Values are mean ± SEM.

(b) A2780 ‘recipient’ cells were pre-treated for 72 hr with exosomes collected from H1299-p53$^{-/-}$, or H1299-p53^{R273H} ‘donor’ cells as for (a), or were left untreated. Recipient cells were then trypsinised and re-plated. 72 hr following re-plating, recycling of integrin α5β1 and TfnR was determined.

(c) Exosomes from H1299-p53$^{-/-}$, or H1299-p53^{R273H} were adjusted to the indicated concentrations and then incubated with recipient H1299-p53$^{-/-}$ cells for 72 hr. Recipient cells were re-plated and grown to confluence over 24hr. Monolayers were wounded with a plastic pipette tip and the speed of their migration into the scratch-wounds determine using time lapse microscopy and cell tracking. Values are mean ± SEM.

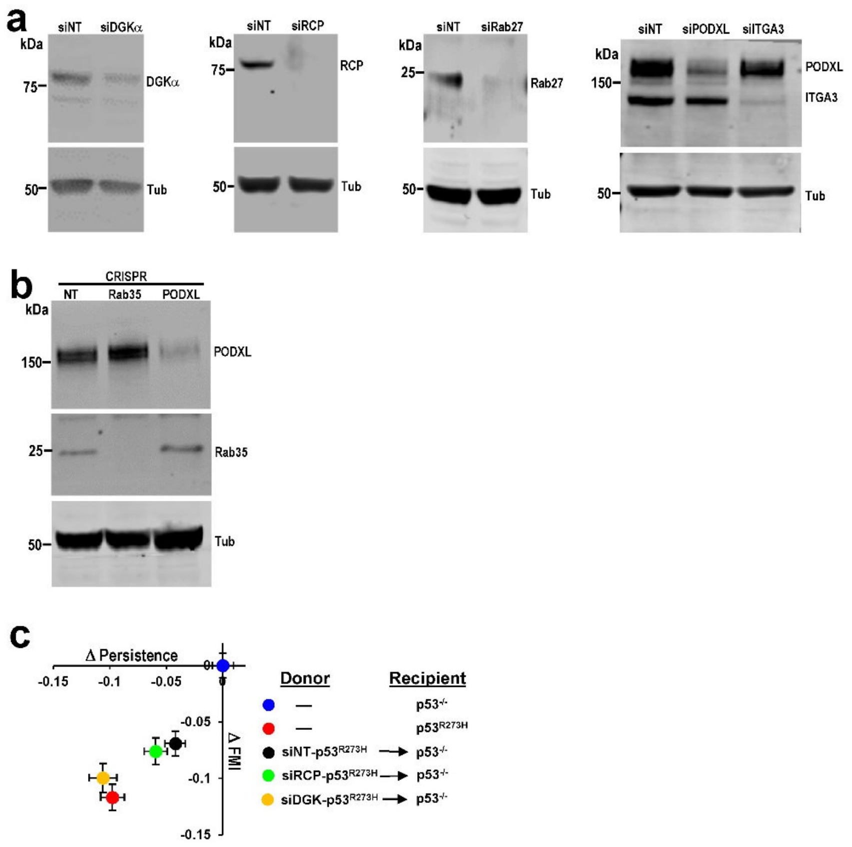

Supplementary Figure 4 Knockdown of trafficking regulators and cargoes

(a) H1299 cells were transfected with SMARTPool siRNA oligonucleotides targeting DGKα (siDGKα), RCP (siRCP), Rab27a and Rab27b (siRab27), Rab35 (siRab35), PODXL (siPODXL), integrin α3 (siITGA3) or a non-targeting control (si-NT). 72 hr following transfection, cells were lysed and the levels of the indicated proteins determined by immunoblotting with tubulin as a loading control.

(b) H1299-p53^{273H} cells were transduced with lentiCRISPR vector to achieve CRISPR-Cas9 mediated disruption of Rab35 or PODXL. Knockdown of Rab35 and PODXL was confirmed by Western blotting with tubulin as a loading control.

(c) Donor H1299-p53^{R273H} cells were transfected with siRNAs targeting RCP (siRCP), DGKα (siDGK), or a non-targeting control (siNT). Exosomes collected from these cells were used to treat H1299-p53$^{-/-}$ recipient cells for 72 hours before their migration characteristics into scratch-wounds was determined as for Fig. S2b. Values are mean ± SEM; n>262 cells.

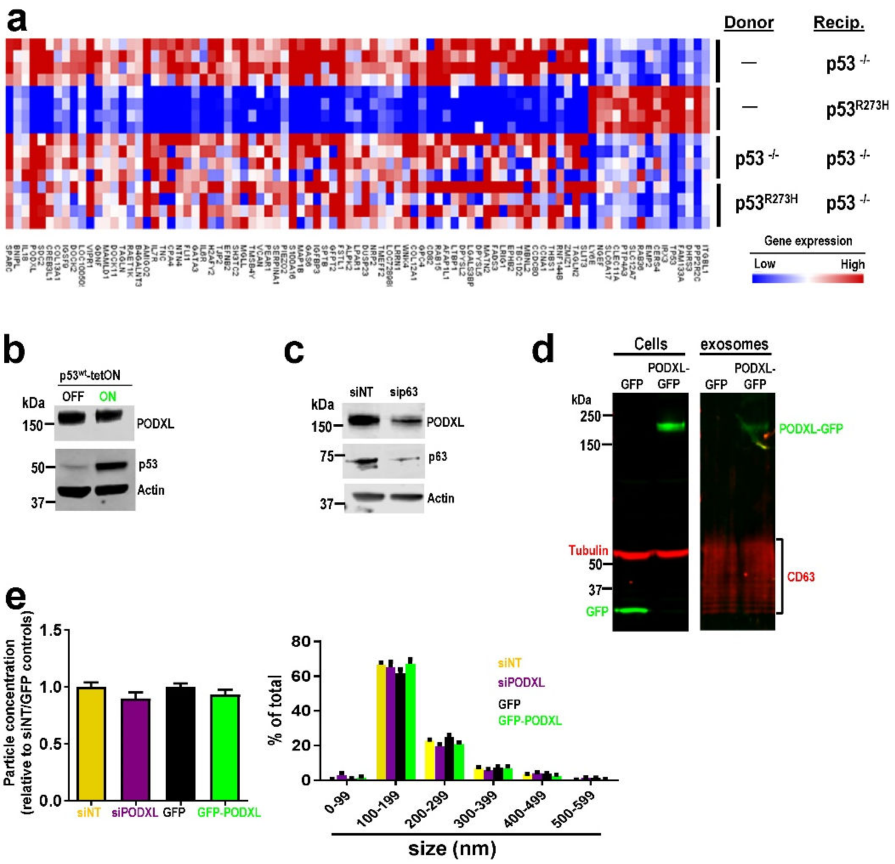

Supplementary Figure 5 Influence of mutp53 PODXL expression

(a) Exosomes from H1299-p53$^{-/-}$ and H1299-p53^{R273H} donor cells were incubated with H1299-p53$^{-/-}$ recipient cells for 72 hr. Recipient cells were re-plated and grown for a further 48 hr prior to lysis. Donor and recipient cells were analysed using RNAseq. These data are extracted from supplementary spreadsheet 2.

(b) H1299-p53tetON cells were incubated in the presence or absence of doxycyclin and cellular levels of wild-type p53 and PODXL were determined by Western blotting.

(c) H1299-p53$^{-/-}$ cells were transfected with siRNAs targeting p63 (sip63) or a non-targeting control (siNT). 48 hr following transfection, the levels of PODXL and p63 were determined by Western blotting.

(d) H1299-p53^{R273H} cells were transfected with PODXL-GFP or GFP. Conditioned media was collected from these cells and exosomes purified from these using differential centrifugation. The levels of PODXL-GFP (green) and GFP (red) in these cells and the exosomes from them were determined by Western blotting. Tubulin and CD63 were used as loading controls for the cell extracts and exosome preparations respectively.

(e) H1299-p53^{R273H} cells were transfected with siRNAs targeting PODXL (siPODXL), a non-targeting control (siNT), GFP or PODXL-GFP. Exosomes were collected from these cells and Nanosight particle tracking used to determine their particle concentration and size distribution. Values are mean ± SEM. n=3.

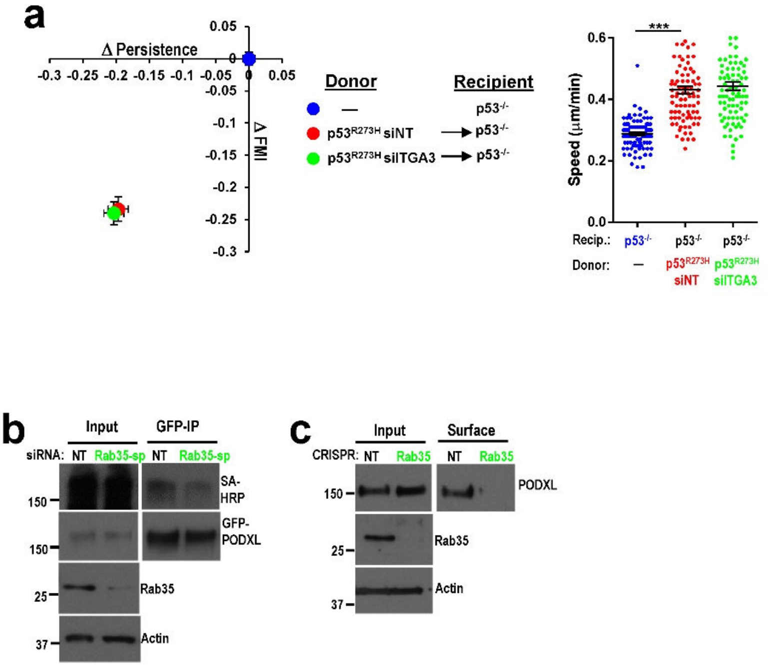

Supplementary Figure 6 Effect of α3β1 knockdown on cell migration and influence of Rab35 on PODXL surface expression

(a) H1299-p53^{273H} donor cells were transfected with siRNAs targeting α3 integrin (siITGA3) or a non-targeting control (siNT). Exosomes were collected from these donor cells and incubated with H1299-p53$^{-/-}$ recipient cells for 72 hr. Recipient cells were re-plated and characteristics of their migration into scratch-wounds determined as for Fig. 2b.

(b) H1299-p53^{R273H} cells were transfected with GFP-PODXL in combination with SMARTPool siRNAs targeting Rab35 (Rab35-sp) or a non-targeting control (NT). Cells were then treated with NHS-Biotin at 4^{0}C to label proteins exposed at the plasma membrane. Labelled cells were lysed, and GFP-PODXL immunoprecipitated using magnetic beads coupled to an antibody recognising GFP (GFP-IP). Biotinylated GFP-PODXL was then visualised by Western blotting with labelled streptavidin. Actin was used as loading control.

(c) H1299 cells in which Rab35 had been disrupted by CRISPR or non-targeting guide sequences (NT) were used. Cells were then treated with NHS-Biotin at 4^{0}C to label proteins exposed at the plasma membrane. Labelled cells were lysed, and labelled proteins precipitated using streptavidin beads. Surface-labelled (surface) and total (input) PODXL were then visualised by Western blotting with antibodies recognising PODXL. Actin was used as loading control.

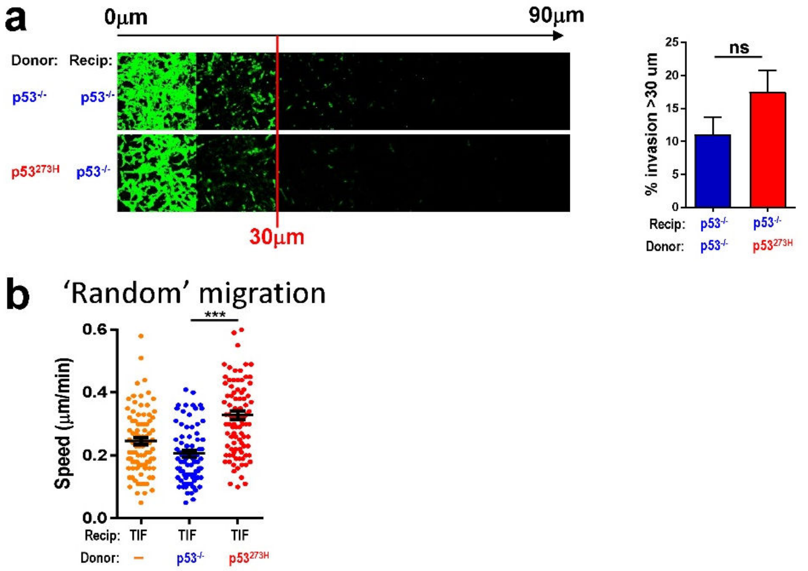

c

PDCL	Class	Variant Type	Start	End	nt Change	AA Change
SQ1	Squamous	Missense_Mutation	7577570	7577570	711C>T	M237I
SQ2	Squamous	Nonsense_Mutation	7579315	7579315	372G>T	C124*
SQ3	Squamous	Frame_Shift_Ins	7578474	7578475	455->G	P152P

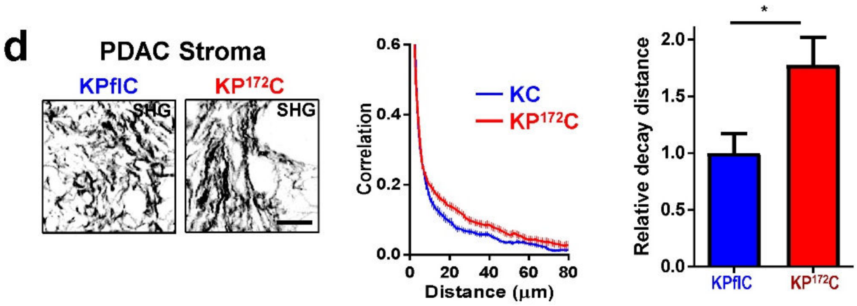

Supplementary Figure 7 Invasion of exosome-treated H1299 cells into Matrigel, random migration of fibroblasts and SHG analysis of PDAC stroma

(a) $p53^{-/-}$ H1299 cells were treated with exosomes collected from $p53^{-/-}$ or $p53^{R273H}$ expressing H1299 cells before being plated onto a Transwell membrane for an inverted invasion assay. Recipient cells were allowed to invade into Matrigel plug supplemented with 25 μg/ml fibronectin for 48hr towards a gradient of EGF (30 ng/ml) and FCS (10%). Invading cells were stained with Calcein-AM, and visualised by confocal microscopy. Serial optical sections were captured at 15 μm intervals, and are presented as a sequence in which the individual optical sections are placed alongside one another with increasing depth from left to right as indicated. Migration was quantitated by measuring the fluorescence intensity of cells penetrating the plug to depths of 30 μm and greater, and expressing this as a percentage of the total fluorescence intensity of all cells within the plug. Values are mean±SEM, n=7.

(b) Telomerase immortalised fibroblasts (TIFs) were treated for 72 hr with exosomes collected from $p53^{-/-}$ or $p53^{R273H}$ expressing H1299 cells. Exosome-treated TIFs were trypsinised and then re-plated at low density onto plastic surfaces and their migration speed determined using time-lapse videomicroscopy following by cell tracking. Values are mean±SEM, n>80 cell precondition, ***$p<0.001$ Mann-Whitney test.

(c) Summary of mutations found in the coding sequence of p53 in the SQ1, SQ2 and SQ3 patient-derived cells lines (PDCLs) from the squamous subtype of pancreatic adenocarcinoma.

(d) $KP^{172}C$ (Pdx1-Cre:KrasG12D/+:p53R172H/+) or $KP^{fl}C$ (Pdx1-Cre:KrasG12D/+:p53fl/+) mice were sacrificed by IP injection of pentobarbital, and the stromal regions of PDAC analysed by SHG microscopy as for Fig. 7(a). Representative SHG pictures of the tumour stroma are displayed (left panel). Bar, 50 µm. Fibrillar collagen organisation was determined using GLCM. The decay curves from these are presented in the centre panel. Weighted means of the decay distances derived from decay curves are displayed in the right panel. Values are weighted mean ± SEM. N=4 animals per condition. * is $p<0.05$, Mann-Whitney.

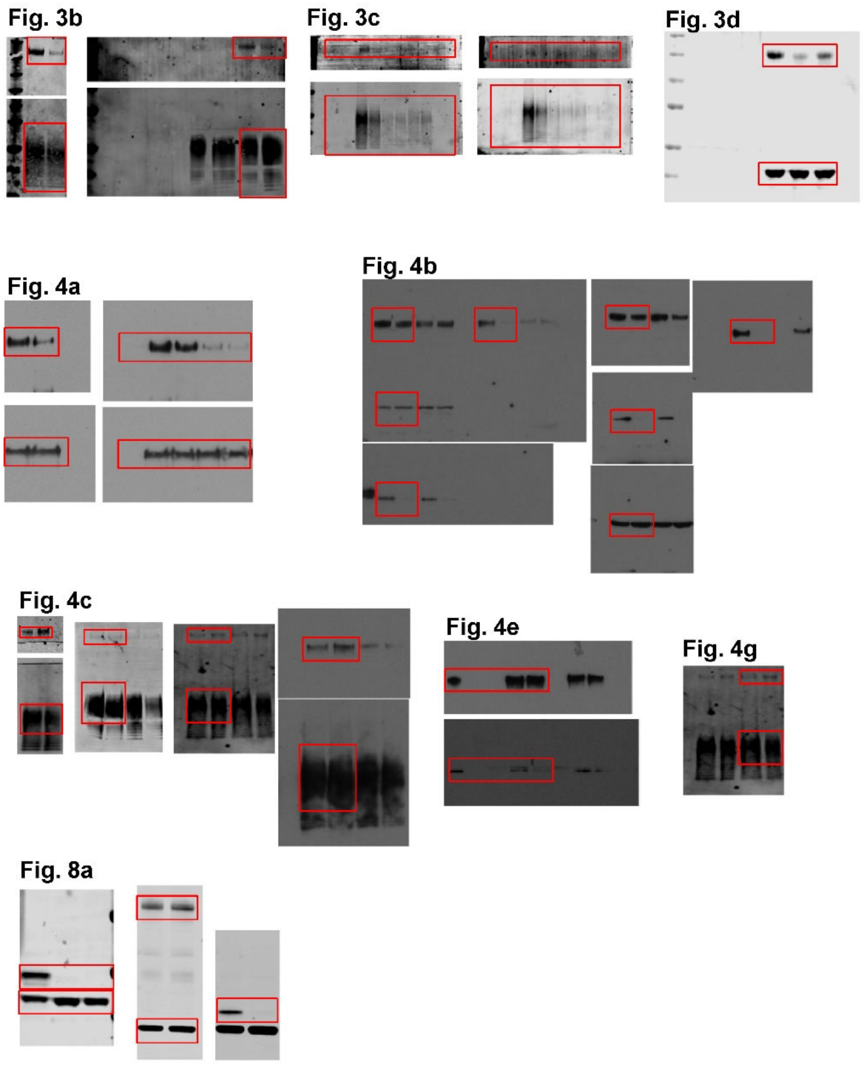

Supplementary Figure 8: Uncropped blots for Figures 3, 4 & 8

Uncropped scans for Western blotting displayed in figures 3, 4, & 8, as indicated. Red boxes highlight the regions displayed.
