# Supplementary material for: Mutant p53s generate pro-invasive niches by influencing exosome podocalyxin levels
Source: Nat Commun. 2018 Nov 29;9:5069. doi: 10.1038/s41467-018-07339-y (PMC6265295; doi:10.1038/s41467-018-07339-y)
Supplement: Supplementary file 3 — Description of Additional Supplementary Files [file 41467_2018_7339_MOESM3_ESM.pdf]

Description of Additional Supplementary Files

File Name: Supplementary Data 1
Description: SILAC-based proteomic comparison of H1299-p53-/- and H1299- p53R273H exosomes H1299-p53-/- and H1299-p53R273H cells were SILAC-labelled with heavy (H) and light (L) amino acids respectively. Conditioned media were collected from labelled cells, exosomes purified from these using differential centrifugation, and their proteome analysed by mass spectrometry. Column F indicates the SILAC ratio of p53R273H/H1299-p53-/- exosomes which is calculated from reciprocal of the ratio of H/L peptides. (Significance B statistic test, False discovery rate of 5%, Perseus software).

File Name: Supplementary Data 2
Description: RNAseq analysis of H1299-p53-/- and H1299-p53R273H cells and exosome-treated H1299-p53-/- cells Exosomes from H1299-p53-/- and H1299-p53R273H donor cells were incubated with H1299- p53-/- recipient cells for 72 hr. Recipient cells were re-plated and grown for a further 48 hr prior to lysis. Donor and recipient cells were analysed using RNAseq. Columns G-J are H1299-p53-/-; columns K-N are H1299-mp53R273H. Columns O-R are H1299-p53-/- recipient cells treated with exosomes from H1299-p53-/- donors; columns S-V are H1299- p53-/- recipient cells treated with exosomes from H1299-p53R273H donors. The ratio of the averages of K-N/G-J are presented in column X. The ratio of the averages of S-V/O-R are presented in column Z.
